# Virus-specific antibodies allow viral replication in the marginal zone, thereby promoting CD8^+^ T-cell priming and viral control

**DOI:** 10.1038/srep19191

**Published:** 2016-01-25

**Authors:** Vikas Duhan, Vishal Khairnar, Sarah-Kim Friedrich, Fan Zhou, Asmae Gassa, Nadine Honke, Namir Shaabani, Nicole Gailus, Lacramioara Botezatu, Cyrus Khandanpour, Ulf Dittmer, Dieter Häussinger, Mike Recher, Cornelia Hardt, Philipp A. Lang, Karl S. Lang

**Affiliations:** 1 Institute of Immunology of the University Hospital in Essen, Medical Faculty, University of Duisburg-Essen, Hufelandstrasse 55, Essen 45147, Germany; 2 Department of Cardiothoracic Surgery, Cologne University, Heart Center, Kerpener strasse 62, 50937 Cologne, Germany; 3 Department of Hematology of the University Hospital in Essen, Medical Faculty, University of Duisburg-Essen, Hufelandstrasse 55, Essen 45147, Germany; 4 Institute of Virology of the University Hospital in Essen, Medical Faculty, University of Duisburg-Essen, Hufelandstrasse 55, Essen 45147, Germany; 5 Clinic of Gastroenterology, Hepatology and Infectious Diseases, Heinrich-Heine-University, Moorenstrasse 5, Düsseldorf 40225, Germany; 6 Clinic for Primary Immunodeficiency, Medical Outpatient Unit and Immunodeficiency Laboratory, Department of Biomedicine, University Hospital, Basel, Switzerland; 7 Molecular Medicine II, Heinrich-Heine-University, Moorenstrasse 5, Düsseldorf 40225, Germany

## Abstract

Clinically used human vaccination aims to induce specific antibodies that can guarantee long-term protection against a pathogen. The reasons that other immune components often fail to induce protective immunity are still debated. Recently we found that enforced viral replication in secondary lymphoid organs is essential for immune activation. In this study we used the lymphocytic choriomeningitis virus (LCMV) to determine whether enforced virus replication occurs in the presence of virus-specific antibodies or virus-specific CD8^+^ T cells. We found that after systemic recall infection with LCMV-WE the presence of virus-specific antibodies allowed intracellular replication of virus in the marginal zone of spleen. In contrast, specific antibodies limited viral replication in liver, lung, and kidney. Upon recall infection with the persistent virus strain LCMV-Docile, viral replication in spleen was essential for the priming of CD8^+^ T cells and for viral control. In contrast to specific antibodies, memory CD8^+^ T cells inhibited viral replication in marginal zone but failed to protect mice from persistent viral infection. We conclude that virus-specific antibodies limit viral infection in peripheral organs but still allow replication of LCMV in the marginal zone, a mechanism that allows immune boosting during recall infection and thereby guarantees control of persistent virus.

Memory formation after antigen challenge is one of the most important hallmarks of the adaptive immune system^1^; it protects the host from exposure to the original or a slightly modified pathogen^1^. Because of this known memory formation, vaccination with attenuated pathogens has been an important tool for preventing outbreaks of severe pathogen-mediated diseases. In the Western world, the World Health Organization recommends approximately 16 vaccinations^2^, 10 of which are antiviral.

Although virus-specific CD8^+^ T cells are known to contribute to the control of viral infections, all recommended vaccinations are aimed at inducing antibodies against a pathogen^3^,^4^,^5^,^6^,^7^. For example, newly designed vaccines against HIV are intended to specifically activate HIV-specific CD8^+^ T cells^8^. However, to date, CD8^+^ T cell–mediated vaccines have failed to protect the host from persistent infection^9^. Therefore, the role of vaccine-induced virus-specific CD8^+^ T cells in long-term protection is still being debated^10^,^11^,^12^. To know in more detail why several vaccines produce protective antibodies but vaccines against HIV and HCV could not do so far. The mechanistic understanding may help to generate new vaccines in future.

Lymphocytic choriomeningitis virus (LCMV) is a non-cytopathic virus with the ability to persist. The acute strain LCMV-WE is usually controlled within 1 or 2 weeks, primarily by virus-specific CD8^+^ T cells. The functions of B cells against LCMV are important for long-term control of the virus; however, CD8^+^ T cells are necessary for early control of LCMV. Infection with the LCMV-Docile strain leads to exhaustion of CD8^+^ T cells and therefore to persistence of the virus in the host^13^.

Recently we found that antigen-presenting cells (CD169^+^ macrophages and CD11c^+^ dendritic cells) within the marginal zone specifically allow viral replication^14^. Enforced viral replication in the spleen is essential for activating the innate and adaptive immune systems^15^. It is still unknown whether enforced viral replication occurs after vaccination or after secondary infection and whether such replication is involved in immune boosting.

In the study reported here we found that, after systemic recall, infection-specific antibodies allow intracellular replication of the virus in the marginal zone of the spleen but limit the replication of infectious virus in liver, lungs, and kidneys. Upon recall infection with the persistent virus strain LCMV-Docile, spleen-specific viral replication is associated with sufficient priming of CD8^+^ T cells and with viral control. In contrast to specific antibodies, memory CD8^+^ T cells inhibit viral replication in the marginal zone thus fail to protect mice against persistent infection.

## Results

### Replication of LCMV in the marginal zone is associated with immune activation and viral control

**Figure 1:**
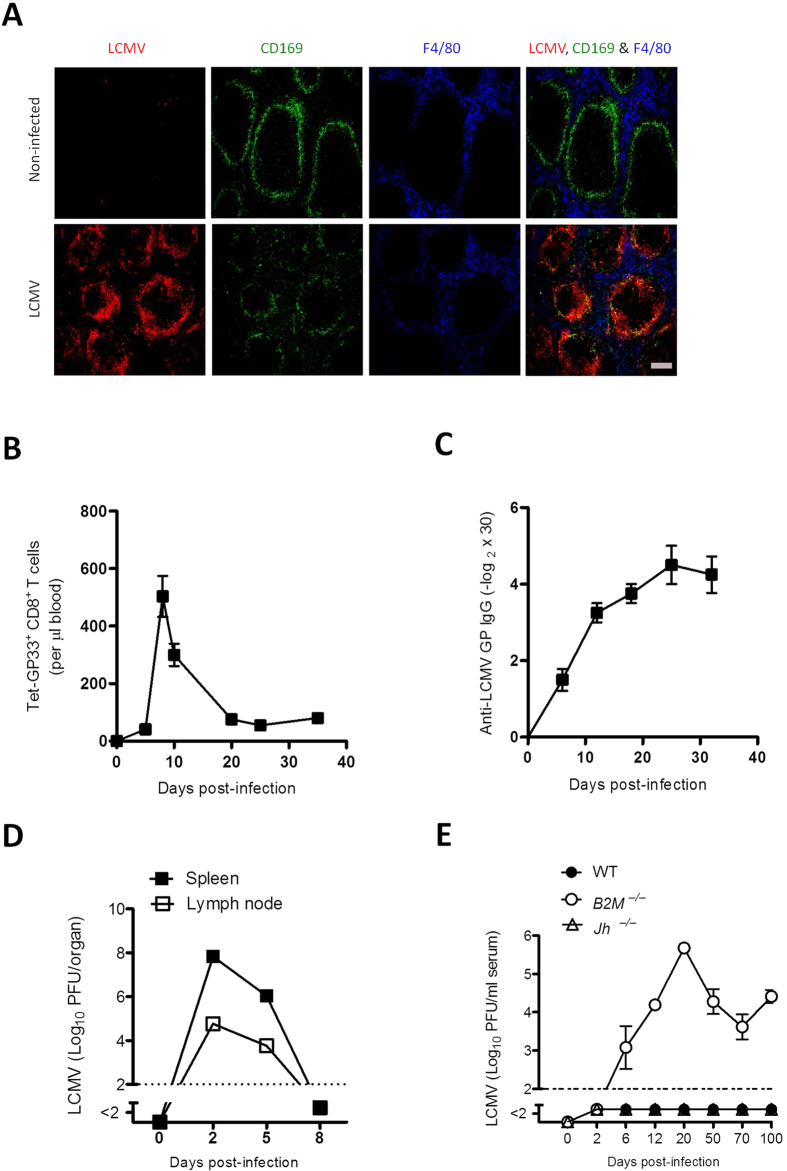
Replication of lymphocytic choriomeningitis virus (LCMV) in the marginal zone is associated with immune activation and viral control C57BL/6 mice were infected intravenously with 2 × 10^4^ plaque-forming units (PFU) of LCMV strain WE (LCMV-WE) and were analysed for various parameters.(**A**) Representative immunofluorescence of spleen after 3 days of infection, stained for LCMV nucleoprotein (red), marginal zone macrophages (CD169, green), and red pulp macrophages (F4/80, blue). One slide representative of 6 slides is shown. Scale bar, 200 μm. (**B**) Total number of LCMV-specific T cells in the blood that were positive for the MHC class I tetramer of the glycoprotein of LCMV (Tet-GP33^+^) and for CD8 (CD8^+^), as measured by fluorescence-activated cell sorting (FACS) at the indicated days after infection (n = 3–7). (**C**) LCMV GP-specific antibodies in serum were analysed by enzyme-linked immunosorbent assay (ELISA) on various days after infection (n = 4). (**D**) Viral titers from spleen and inguinal lymph nodes were analysed by plaque-forming assay at the indicated time points after infection (*n* = 3). (**E**) Viral titers in serum of wild type (WT), *B2m*^*−/−*^, and *Jh*^*−*^/^*−*^ mice on various days after infection (*n* = *4*). Horizontal dotted lines designate the detection limit. Data are shown as mean ± SEM.

During primary viral infection, LCMV replicates in the marginal zone; this replication is essential for inducing adaptive immunity against the virus^15^. Histologic examination of the spleen on day 3 after infection with 2 × 10^4^ plaque-forming units (PFU) of the acute strain LCMV-WE detected staining of LCMV along the marginal zone (Fig. 1A). This finding was associated with the induction of virus-specific CD8^+^ T cells (Fig. 1B) and the induction of LCMV-specific antibodies (Fig. 1C); these activities resulted in control of the virus within 8 days (Fig. 1D). For early control of the virus, virus-specific CD8^+^ T cells are essential, as demonstrated by our finding that *B2m*^−/−^ mice, which lack CD8^+^ T cells, could not control the virus in the circulation (Fig 1E). *Jh*^−/−^ mice, which are deficient in B cells, controlled the virus in a manner similar to that of wild-type mice (Fig 1E); this finding emphasizes that early control of LCMV-WE depends primarily on virus-specific CD8^+^ T cells. Therefore, we conclude that enforced viral replication leads to the priming of CD8^+^ T cells, which are necessary for early viral control, whereas B cells are most likely needed for long-term protection against LCMV^16^,^17^.

### Virus-specific antibodies, but not virus-specific CD8^+^ T cells, allow viral replication in the marginal zone

**Figure 2:**
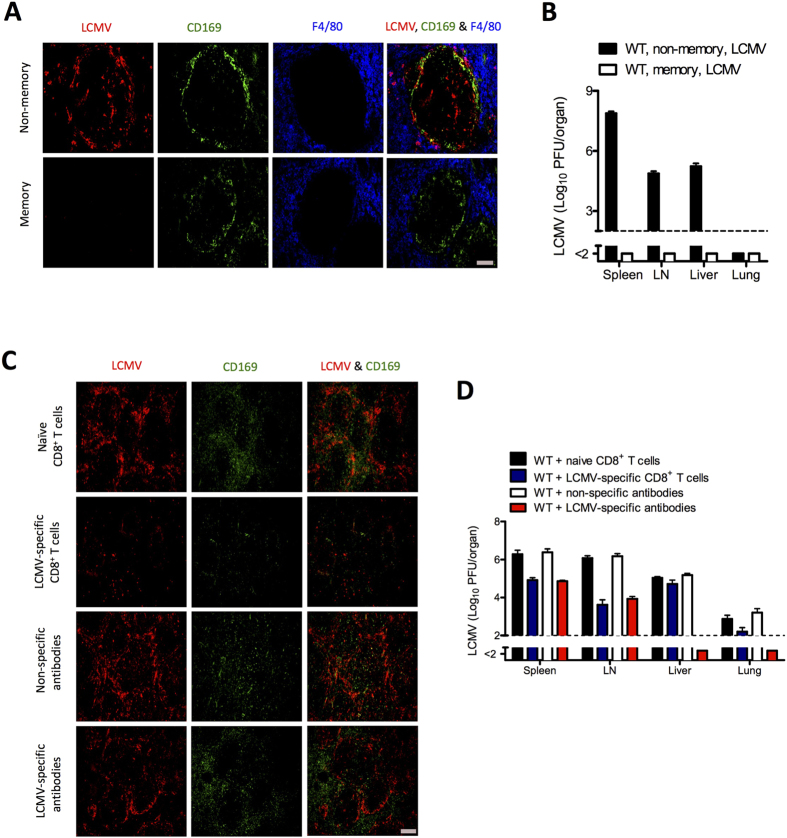
Virus-specific antibodies, but not virus-specific CD8^+^ T cells, allow viral replication in the marginal zone. (**A**) Naïve (non-memory) and memory C57BL/6 mice were infected intravenously with 2 × 10^7^ plaque-forming units (PFU) of lymphocytic choriomeningitis virus strain WE (LCMV-WE). After day 1 spleen sections were stained for LCMV nucleoprotein (red), marginal zone macrophages (CD169, green), and red pulp macrophages (F4/80, blue). One slide representative of 4 slides is shown. Scale bar, 100 μm. (**B**) Viral titers from spleen, inguinal lymph nodes (LN), liver, and lungs of naïve and memory C57BL/6 mice infected intravenously with 2 × 10^7^ PFU of LCMV-WE, as measured on day 1 (n = 4–6). (C, D) C57BL/6 naïve mice were injected separately with naïve CD8^+^ T cells and non-specific antibodies (naïve serum) collected from naïve mice, and with LCMV-specific CD8^+^ T cells and LCMV-specific antibodies (immune serum) collected from memory mice. After 2 days all mice were infected with 2 × 10^6^ PFU of LCMV-WE. (**C**) Representative immunofluorescence of spleen after 3 days of viral infection, stained for LCMV nucleoprotein (red) and marginal zone macrophages (CD169, green). One slide representative of 6 slides is shown. Scale bar, 200 μm. (**D**) Viral titers from spleen, inguinal lymph node (LN), liver, and lungs after 3 days of viral infection (n = 6–7). Horizontal dotted lines designate the detection limit. Data are shown as mean ± SEM and are pooled from 2 or 3 independent experiments.

Recall viral infections often boost the existing immune response^18^. Whether an immune-response boost after a recall infection with LCMV requires viral replication and whether adaptive memory components allow replication of virus in the marginal zone remain unknown. To gain insights into this question we first infected mice with 200 PFU of LCMV-WE and then 50 days later challenged them with 2 × 10^7^ PFU of LCMV-WE. We could not detect any replication of virus within the marginal zone after recall infection (Fig. 2A). In line with this finding, no infectious virus was detected in any organ tested (Fig. 2B). These findings suggest that memory mice are well protected against LCMV recall infection.

Next we aimed to determine how various specific memory immune components limit viral replication in the marginal zone and peripheral organs. We infected wild-type (WT) mice with 2 × 10^6^ PFU of LCMV-WE, and after 80 to 120 days of infection we transferred various memory components from these infected mice into naïve WT mice. For control mice we transferred immune components from naïve mice to naïve mice. We focused on the transfer of serum for virus-specific antibodies, sorted splenic B cells, splenic CD8^+^ T cells, and splenic CD4^+^ T cells. For each memory component we transferred approximately 20% of the specific compartment of an LCMV-infected mouse (see Material and Methods). Two days after transfer we challenged mice with 2 × 10^6^ PFU of LCMV-WE, and at days 1, 2, and 3 we analyzed viral distribution. Mice that received naïve immune components exhibited normal staining of LCMV in the marginal zone (Fig. 2C, supplementary Figure 1A). Both virus-specific CD8^+^ T cells and antibodies allowed replication of virus at day 1 (Supplementary Figure 1A and 1B). After day 1, virus-specific CD8^+^ T cells inhibited the replication of virus in the marginal zone (Fig. 2C, Supplementary Figure 1A). Perforin deficient LCMV-specific CD8^+^ T cells which were primed with recombinant LCMV (rLCMV) (see material and methods) did not effect the virus replication in marginal zone (Supplementary Figure 1C) suggesting that direct cytotoxicity of virus-specific CD8^+^ T cells mediated by perforin killed virus-infected antigen-presenting cells in the marginal zone (Supplementary Figure 1C). Transfer of virus-specific antibodies slightly reduced LCMV staining in the marginal zone but still allowed abundant replication at any time tested (Fig. 2C, Supplementary Figure 1A). Transfer of memory CD4^+^ T cells or memory B cells exerted no measurable influence on the replication of LCMV in the marginal zone (Supplementary Fig. 2A).

Next we analyzed the role of virus-specific CD8^+^ T cells and virus-specific antibodies on the early distribution of virus in other organs. Virus-specific CD8^+^ T cells reduced infectious virus in the spleen and lymph node alone after day 1 (Fig. 2D and Supplementary Figure 1B). Interestingly, although we found limited staining of virus-infected cells in the marginal zone, the levels of infectious LCMV were still easily detectable. This is probably due to the fact that virus-specific CD8^+^ T cells target virus-bearing cells rather than free infectious virus. In peripheral organs, virus-specific CD8^+^ T cells exerted only a limited effect on viral replication (Fig. 2D and Supplementary Figure 1B). This finding suggests that virus-specific CD8^+^ T cells exert limited influence on the early replication of virus in the spleen, lymph nodes, liver, and lungs.

Like virus-specific CD8^+^ T cells, virus-specific antibodies reduced the amount of infectious virus in the spleen and lymph nodes but still allowed replication of virus (Fig. 2D, Supplementary Figure 1B). However, unlike virus-specific CD8^+^ T cells, virus-specific antibodies completely blunted the replication of virus in all peripheral organs tested (Fig. 2D, Supplementary Figure 1B). Memory B cells and CD4^+^ T cells exerted no significant effect on the replication of virus in any of the organs tested (Supplementary Figure 2B), a finding implying that memory B and CD4^+^ T cells have no impact on the early distribution of virus. Therefore, we conclude that virus-specific antibodies allow the replication of virus in the splenic marginal zone but protect against the replication of virus in peripheral organs. Virus-specific CD8^+^ T cells inhibit the replication of virus in the marginal zone but have limited impact on the replication of virus in peripheral organs.

**Figure 3:**
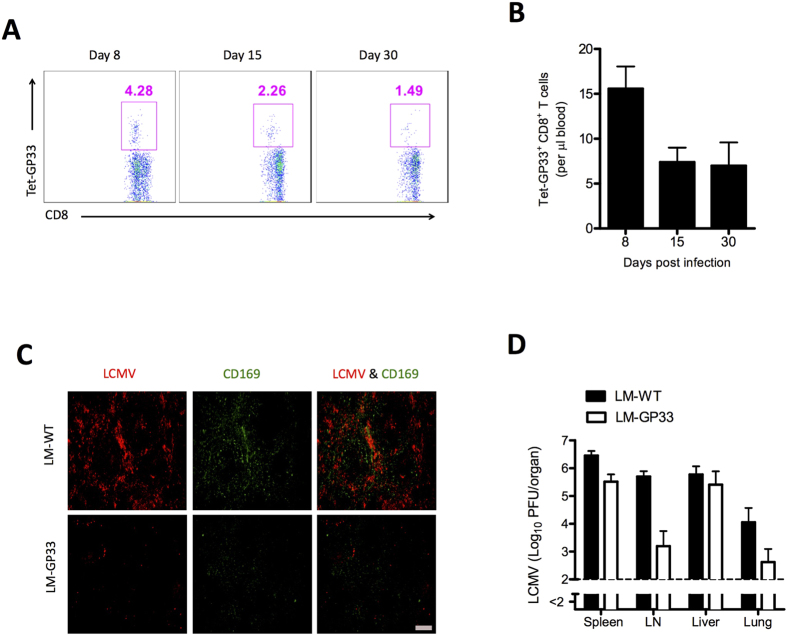
Inhibition of viral replication in splenic marginal zone of mice primed with recombinant *Listeria monocytogenes* expressing the glycoprotein of LCMV C57BL/6 naïve mice were infected with 1 × 10^6^ colony-forming units (CFU) of *Listeria monocytogenes* expressing the glycoprotein of lymphocytic choriomeningitis virus (LM-GP33), and control mice were infected with a lower dose (1 × 10^4^ CFU) of wild-type *L. monocytogenes* (LM-WT) as higher dose is lethal for mice. After 30 days mice were injected with 2 × 10^6^ PFU of LCMV-WE. (**A**) Fluorescence-activated cell sorting (FACS) plots showing the frequency of T cells that were positive for the MHC class I tetramer of the glycoprotein of LCMV (Tet-GP33^+^) and for CD8 (CD8^+^) in the total number of CD8^+^ T cells in the blood at indicated days after LM-GP33 infection. (**B**) Graph showing the total number of Tet-GP33^+^ CD8^+^ T cells in blood on various days after LM-GP33 infection (n = 6). (**C**) Immunohistochemical analysis of spleens from LM-GP33–primed mice after 3 days of LCMV strain WE (LCMV-WE) infection, showing LCMV nucleoprotein (red) and marginal zone macrophages (CD169, green). Scale bar, 200 μm (n = 5). (**D**) Viral titers from spleen, inguinal LN, liver, and lungs after 3 days of LCMV-WE infection (n = 5). Horizontal dotted lines designate the detection limit. Data are shown as mean ± SEM and are pooled from 2 independent experiments.

Next we determined whether antigen-specific CD8^+^ T cells that were primed with *Listeria monocytogenes* behaved in the same manner as transferred virus-specific CD8^+^ T cells. We infected WT mice with *L. monocytogenes* expressing the glycoprotein of LCMV (LM-GP33) or with wild-type *L. monocytogenes* (LM-WT). Mice infected with LM-GP33 generated LCMV GP33-specific CD8^+^ T cells (Fig. 3A and B). After 30 days the mice were infected with LCMV-WE. Control mice infected with LM-WT exhibited normal replication of virus in the marginal zone (Fig. 3C). In contrast, mice challenged with LM-GP33 did not exhibit viral staining in the marginal zone (Fig. 3C), a finding indicating inhibition of virus in the marginal zone by virus-specific CD8^+^ T cells. Virus-specific CD8^+^ T cells generated after LM-GP33 infection reduced the replication of infectious virus in lymph nodes and lungs; however, they did not influence the replication of virus in the liver (Fig. 3D).

Therefore, we conclude that virus-specific antibodies allow viral replication in the marginal zone but suppress viral replication in other organs. Virus-specific CD8^+^ T cells suppress viral replication in the marginal zone but have limited influence on viral replication in peripheral organs.

### Virus-specific antibodies allow innate and adaptive immune activation

**Figure 4:**
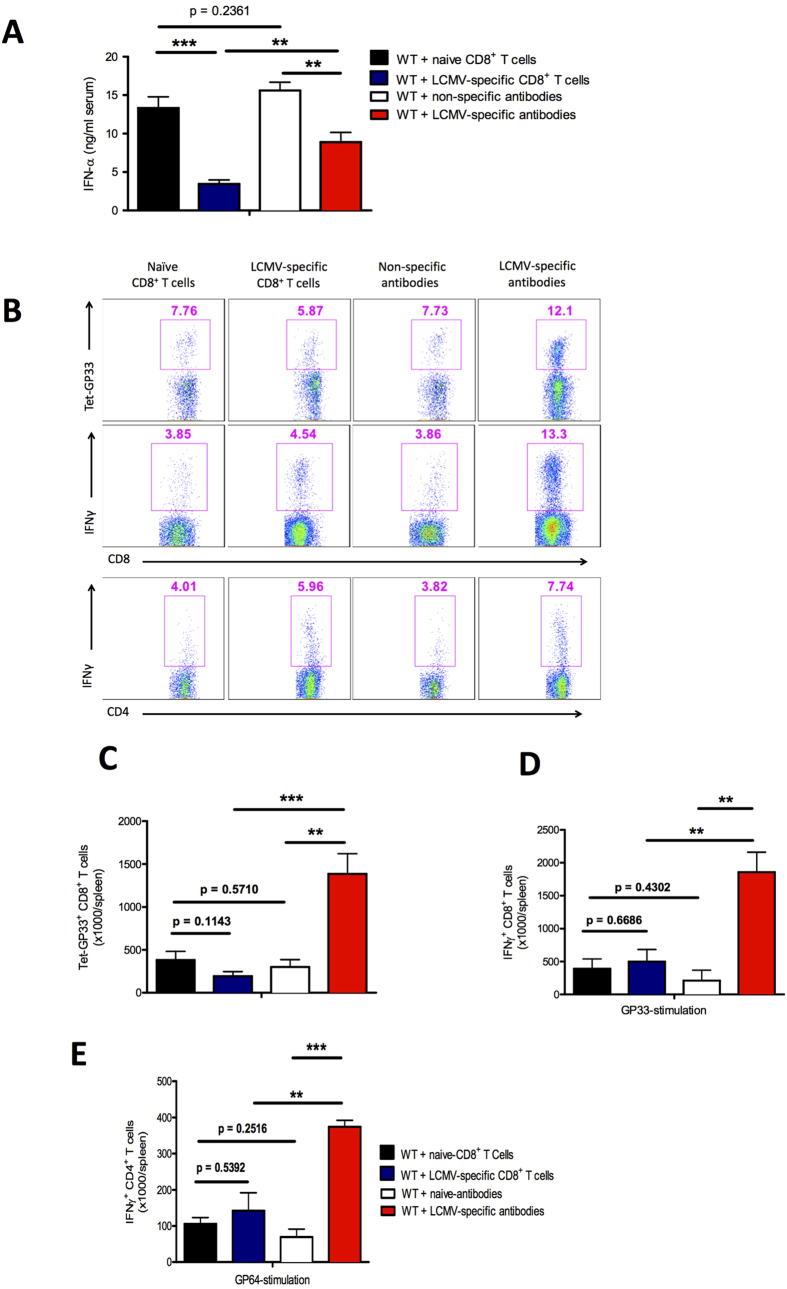
Virus-specific antibodies allow innate and adaptive immune activation C57BL/6 naïve mice were injected separately with naïve CD8^+^ T cells and non-specific antibodies (naïve serum) collected from naïve mice, and with lymphocytic choriomeningitis (LCMV)-specific CD8^+^ T cells and LCMV-specific antibodies (immune serum) collected from memory mice. After 2 days all mice were infected with 2 × 10^6^ plaque-forming units (PFU) of LCMV strain WE (LCMV-WE). (**A**) Levels of interferon (IFN)-α were measured in the serum by enzyme-linked immunosorbent assay (ELISA) after 2 days of infection (n = 6). (**B**) Representative fluorescence-activated cell sorting (FACS) plots showing the frequency of LCMV-specific T cells in the spleen that were positive for the MHC class I tetramer of the glycoprotein of LCMV (Tet-GP33^+^) and for CD8 (CD8^+^) in the total number of CD8^+^ T cells in the spleen (upper plots). Frequency of IFN-γ^+^ CD8^+^ T cells (middle plots) and IFN-γ^+^ CD4^+^ T cells (lower plots) in the spleen after *in vitro* stimulation with LCMV GP33 and LCMV GP64 peptide respectively after 10 days of viral infection. (**C**) Total number of LCMV-specific Tet-GP33^+^ CD8^+^ T cells in the spleen after 10 days of viral infection (n = 4–6). (**D**) Total number of IFN-γ^+^ CD8^+^ T cells in the spleen after 10 days of viral infection and after *in vitro* stimulation with LCMV GP33 peptide for 5 hours (n = 4–6). (**E**) Total number of IFN-γ–producing CD4^+^ T cells in the spleen after 10 days of viral infection and after *in vitro* stimulation with LCMV GP64 peptide for 5 hours (n = 3–6). Data are shown as mean ± SEM and are pooled from 2 or 3 independent experiments. *P < 0.05; **P < 0.01; ***P < 0.001 (Student’s *t*-test).

We found that virus-specific CD8^+^ T cells and virus-specific antibodies exert different effects on early viral distribution. Next we examined how differences in viral replication affect innate and adaptive immune activation. We transferred virus-specific antibodies or CD8^+^ T cells from memory mice into naïve C57BL/6 mice and infected them with LCMV-WE. Virus-specific CD8^+^ T cells strongly reduced the induction of antiviral interferon type I (IFN-I) (Fig. 4A). The presence of virus-specific antibodies also reduced the IFN-I response (Fig. 4A) but to a lower extent than did virus-specific CD8^+^ T cells (Fig. 4A).

Next we examined the antiviral CD8^+^ T cell response after LCMV infection. Transfer of virus-specific CD8^+^ T cells before infection exerted no effect on the total number of virus-specific CD8^+^ T cells at day 10 after challenge with LCMV-WE (Fig. 4B upper panel, 4C). Non-transferred endogenous virus-specific CD8^+^ T cells exhibited reduced activation in the presence of memory CD8^+^ T cells (Supplementary Figure 3). In contrast, in the presence of virus-specific antibodies the expansion of virus-specific CD8^+^ T cells was greater than that in mice receiving virus-specific CD8^+^ T cells or in mice receiving non-specific immune components (Fig. 4B upper panel and 4C). In addition, the production of IFN-γ after *in vitro* restimulation was enhanced in splenocytes derived from mice treated with virus-specific antibodies (Fig. 4B middle panel and 4D). The total number of IFN-γ–producing CD4^+^ T cells was also enhanced in the spleens of mice that had received virus-specific antibodies (Fig. 4B lower panel and 4E).

We conclude that virus-specific antibodies allow innate immune activation and exert a positive effect on the induction of virus-specific CD8^+^ T cells. In contrast, the presence of virus-specific CD8^+^ T cells is not beneficial for immune activation upon challenge infection.

### Virus-specific antibodies protect against immunopathology and lead to control of virus

**Figure 5:**
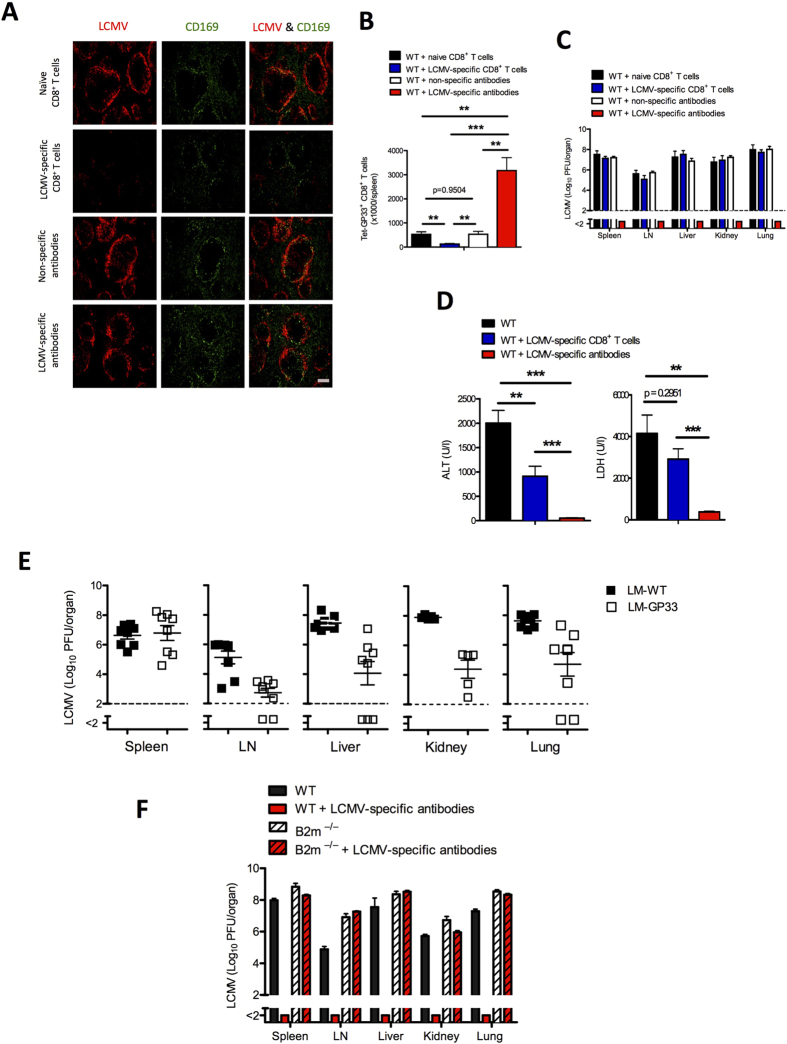
Virus-specific antibodies protect against immunopathology and lead to control of virus C57BL/6 naïve mice were injected separately with naïve CD8^+^ T cells and non-specific antibodies (naïve serum) collected from naïve mice, and with lymphocytic choriomeningitis virus (LCMV)-specific CD8^+^ T cells and LCMV-specific antibodies (immune serum) collected from memory mice. After 2 days all mice were infected with 2 × 10^4^ plaque-forming units (PFU) of LCMV-Docile. (**A**) Representative immunofluorescence of spleen after 3 days of infection, stained for LCMV nucleoprotein (red) and marginal zone macrophages (CD169, green). One slide representative of 3 slides is shown. Scale bar, 200 μm. (**B**) Total number of LCMV-specific T cells in the spleen that were positive for the MHC class I tetramer of the glycoprotein of LCMV (Tet-GP33^+^) and for CD8 (CD8^+^) after 10 days of viral infection (n = 4–7). (**C**) Viral titers from spleen, inguinal LN, liver, kidney, and lungs after 10 days of viral infection (n = 7–10). (**D**) Levels of alanine aminotransaminase (ALT) and lactate dehydrogenase (LDH) in serum were measured after 10 days of viral infection (n = 7–10). (**E**) C57BL/6 mice primed with *Listeria monocytogenes* expressing the glycoprotein of lymphocytic choriomeningitis virus (LM-gp33) were infected with 2 × 10^6^ PFU of LCMV-Docile. After 10 days viral titers were measured in various organs, as indicated (n = 5–8). (**F**) C57BL/6 and *B2m*^−/−^ *mice* were treated with virus-specific antibodies or were left untreated. After 2 days all mice were infected with 2 × 10^4^ PFU of LCMV-Docile. Viral titers from spleen, inguinal LN, liver, kidney, and lungs were measured after 10 days of viral infection (n = 3–4). Horizontal dotted lines designate the detection limit. Data are shown as mean ± SEM and are pooled from 2 or 3 independent experiments. *P < 0.05; **P < 0.01; ***P < 0.001 (Student’s *t*-test).

Next we investigated the influence of virus-specific CD8^+^ T cells or virus-specific antibodies on overall outcome after infection with the persistent LCMV-Docile strain. Unlike LCMV-WE, LCMV-Docile induces persistent viral infection (Supplementary Figure 4A). We transferred virus-specific CD8^+^ T cells and virus-specific antibodies to naïve mice and infected them with 2 × 10^4^ PFU of LCMV-Docile. As was true of challenge infection with the acute LCMV-WE strain, the presence of virus-specific antibodies allowed viral replication, and virus-specific CD8^+^ T cells almost inhibited viral replication in the splenic marginal zone (Fig. 5A). Unlike virus-specific CD8^+^ T cells, virus-specific antibodies totally blunted viral load in the peripheral organs (Supplementary Figure 4B). Transfer of virus-specific antibodies enhanced the priming and expansion of virus-specific CD8^+^ T cells (Fig. 5B). But transfer of virus-specific CD8^+^ T cells before infection abrogated the expansion of antigen specific CD8^+^ T cells (Fig. 5B). In line with these findings, our study showed that mice that received virus-specific antibodies before infection could eliminate LCMV-Docile, whereas it persisted in mice transferred with naïve immune components and in mice treated with virus-specific CD8^+^ T cells (Fig. 5C). Virus-specific antibodies prevented elevation of serum alanine aminotransaminase (ALT) and lactate dehydrogenase (LDH) activity, which are signs of virus-induced immunopathology (Fig. 5D). Interestingly, the transfer of virus-specific CD8^+^ T cells reduced immunopathology, a finding that is in line with reduced activation of CD8^+^ T cells. Next we subjected mice that had been vaccinated with LM-GP33 to infection with LCMV-Docile. As in our transfer experiments, LM-GP33–vaccinated mice could not eliminate LCMV-Docile (Fig. 5E).

Next we examined whether the priming of CD8^+^ T cells in the presence of virus-specific antibodies was essential for the control of LCMV-Docile. We transferred virus-specific antibodies into *B2m*^−/−^ mice, which lack CD8^+^ T cells. In the absence of CD8^+^ T cells, virus-specific antibodies did not control LCMV-Docile infection (Fig. 5F), a finding suggesting that secondary CD8^+^ T-cell activation is essential for viral control after challenge infection.

### Virus-specific antibodies enhance priming and expansion of CD8^+^ T cells

**Figure 6:**
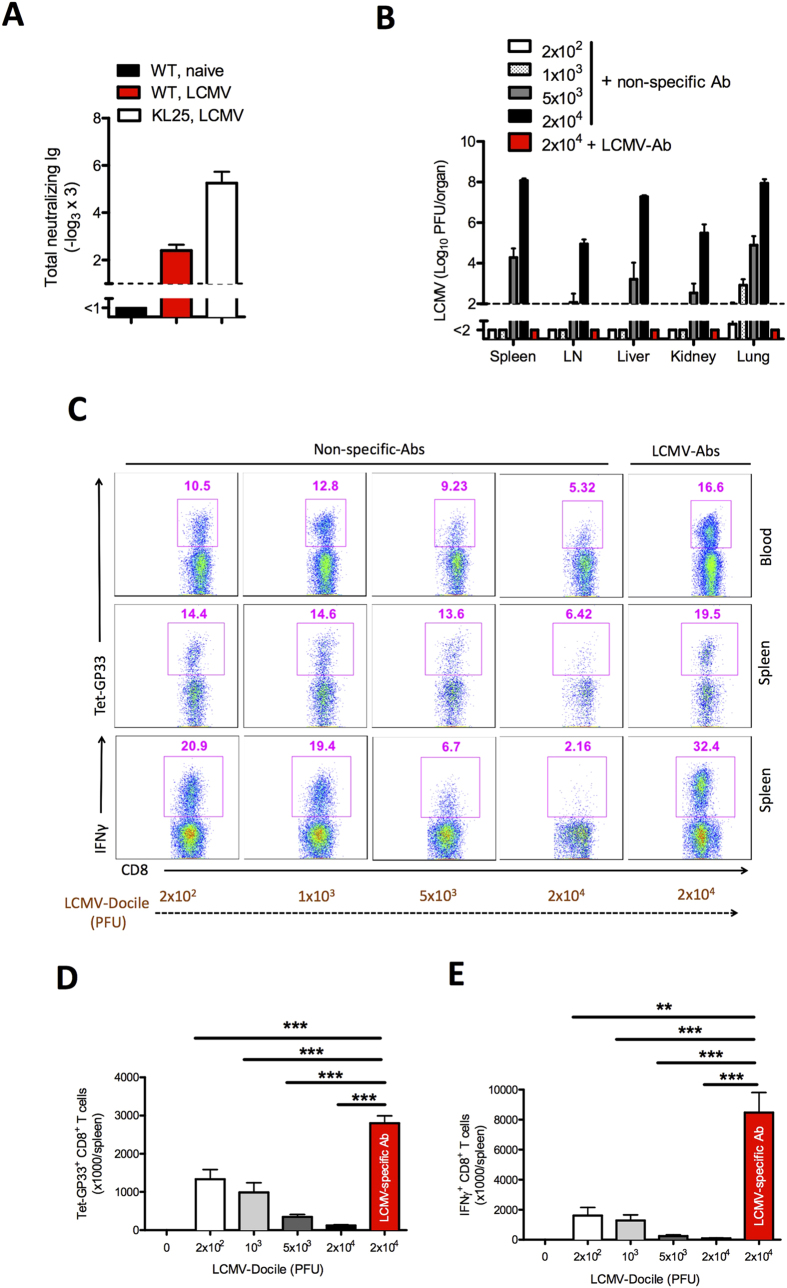
Virus-specific antibodies enhance priming and expansion of CD8^+^ T cells. (**A**) Graph showing the total neutralizing antibodies in naïve serum (WT, naive), serum from memory C57BL/6 mice (WT, LCMV) on day 100 and from KL25 mice (KL25, LCMV) on day 10 after infection with 2 × 10^6^ PFU of LCMV-WE and determined by *in vitro* neutralization assay (n = 4–5). (**B–E**) Naïve C57BL/6 mice were injected with non-specific antibodies or LCMV-specific antibodies. Mice treated with non-specific antibodies were infected separately with 2 × 10^2^, 1 × 10^3^, 5 × 10^3^, or 2 × 10^4^ PFU of LCMV-Docile. Mice treated with LCMV-specific antibodies were infected with 2 × 10^4^ PFU of LCMV-Docile. (**B**) Viral titers in various organs after 10 days of viral infection (n = 6). (**C**) FACS plots representing the frequency of LCMV-specific Tet-GP33^+^ CD8^+^ T cells in blood (upper plots) and spleen (middle plots) after 10 days of infection. Lower plots show the frequency of interferon (IFN)-γ^+^ CD8^+^ T cells in spleen after 10 days of viral infection and *in vitro* stimulation with LCMV GP33 peptide (n = 6–9). (**D**) Total number of LCMV-specific Tet-GP33^+^ CD8^+^ T cells in spleen after 10 days of viral infection (n = 6–9). (**E**) Total number of IFN-γ^+^ CD8^+^ T cells in spleen after *in vitro* stimulation with LCMV GP33 peptide on day 10 of infection (n = 6–9). Horizontal dotted lines designate the detection limit. Data are shown as mean ± SEM and are pooled from 2 or 3 independent experiments. *P < 0.05; **P < 0.01; ***P < 0.001 (Student’s *t*-test).

We found that the priming and expansion of virus-specific CD8^+^ T cells was highly improved in the presence of virus-specific antibodies. Virus-specific antibodies, which were used for the transferred experiments showed only slight neutralization capacity in *in-vitro* assay (Fig. 6A). To gain insights into the mechanism of action of virus-specific antibodies, we first determined whether these antibodies simply reduced the amount of free infectious virus immediately after infection. To do so, we examined CD8^+^ T-cell activation after infection with various doses of LCMV-Docile with or without additional treatment with LCMV-specific antibodies (LCMV-Ab). Viral control was possible after infection with 200 PFU, 1000 PFU, or 20,000 PFU in the presence of virus-specific antibodies (Fig. 6B). In the absence of these antibodies, maximal CD8^+^ T-cell priming was achieved with an infectious dose of 200 PFU LCMV-Docile (Fig. 6C, D and E). In the presence of virus-specific antibodies the priming of CD8^+^ T cells was even higher than after infection of 200 PFU (Fig. 6D and E), a finding suggesting that additional mechanisms other than reducing the infectious dose contribute to enhancements in the priming of CD8^+^ T cells.

### Immune activation in the presence of virus-specific antibodies is essential for controlling persistent infection

**Figure 7:**
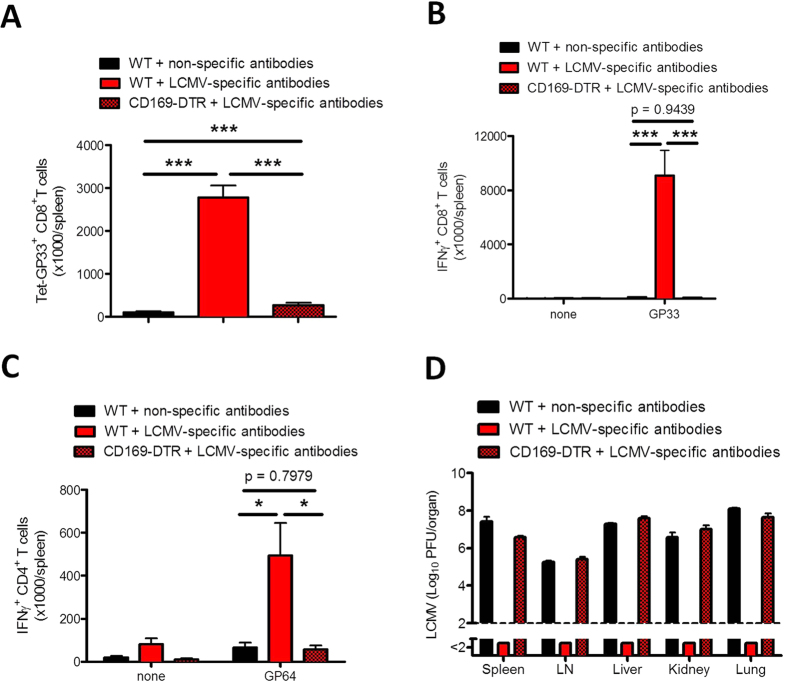
Immune activation in the presence of virus-specific antibodies is essential for controlling persistent viral infection. Naïve C57BL/6 mice and CD169-DTR mice were treated with diphtheria toxin (30 μg/kg) on day -3 and day 2 and were injected with lymphocytic choriomeningitis (LCMV)-specific antibodies on day -2. One group of C57BL/6 mice was injected with non-specific antibodies on day -2. All mice were infected with 2 × 10^4^ plaque-forming units (PFU) of LCMV-Docile on day 0. Ten days later mice were evaluated for various parameters. (**A**) Total number of LCMV-specific T cells in spleen that were positive for the MHC class I tetramer of the glycoprotein of LCMV (Tet-GP33^+^) and for CD8 (CD8^+^) (n = 6). (**B**) Total number of interferon (IFN)-γ^+^ CD8^+^ T cells in spleen was determined after *in vitro* stimulation with or without LCMV GP33 peptide for 5 hours (n = 6). (**C**) Total number of IFN-γ producing CD4^+^ T cells in spleen after *in vitro* stimulation with or without LCMV GP64 peptide for 5 hours (n = 6). (**D**) Viral titers in spleen, inguinal lymph nodes (LN), liver, kidney, and lungs were measured after 10 days of viral infection (n = 6). Data are shown as mean ± SEM and are pooled from 2 independent experiments. *P < 0.05; **P < 0.01 and ***P < 0.001 (Student’s *t*-test).

We found that the transfer of virus-specific antibodies leads to strong priming of CD8^+^ T cells, which is associated with viral control and limited immunopathology. Recently we found that the IFN-I inhibitor *Usp18* is highly expressed in CD169^+^ marginal zone macrophages and dendritic cells and is therefore crucial for viral replication in these cells reside in spleen and lymph nodes^14^,^15^. The absence of either marginal zone macrophages or *Usp18* limits viral replication, and this limitation is associated with the absence of antiviral innate or adaptive immune responses^14^,^15^. To determine whether *Usp18*-dependent viral replication in marginal zone macrophages is also necessary for the activation of CD8^+^ T cells after secondary antigen challenge, we next transferred virus-specific antibodies to WT and CD169-DTR mice, in which specific CD169^+^ marginal zone macrophages can be depleted by injecting diphtheria toxin, and infected them with LCMV-Docile. In the absence of marginal zone macrophages, virus-specific CD8^+^ T-cell expansion (Fig. 7A) and IFN-γ production by CD8^+^ T cells (Fig. 7B) and CD4^+^ T cells (Fig. 7C) were reduced. This lack of a sufficient virus-specific CD8^+^ T-cell response leads to viral persistence (Fig. 7D).

**Figure 8:**
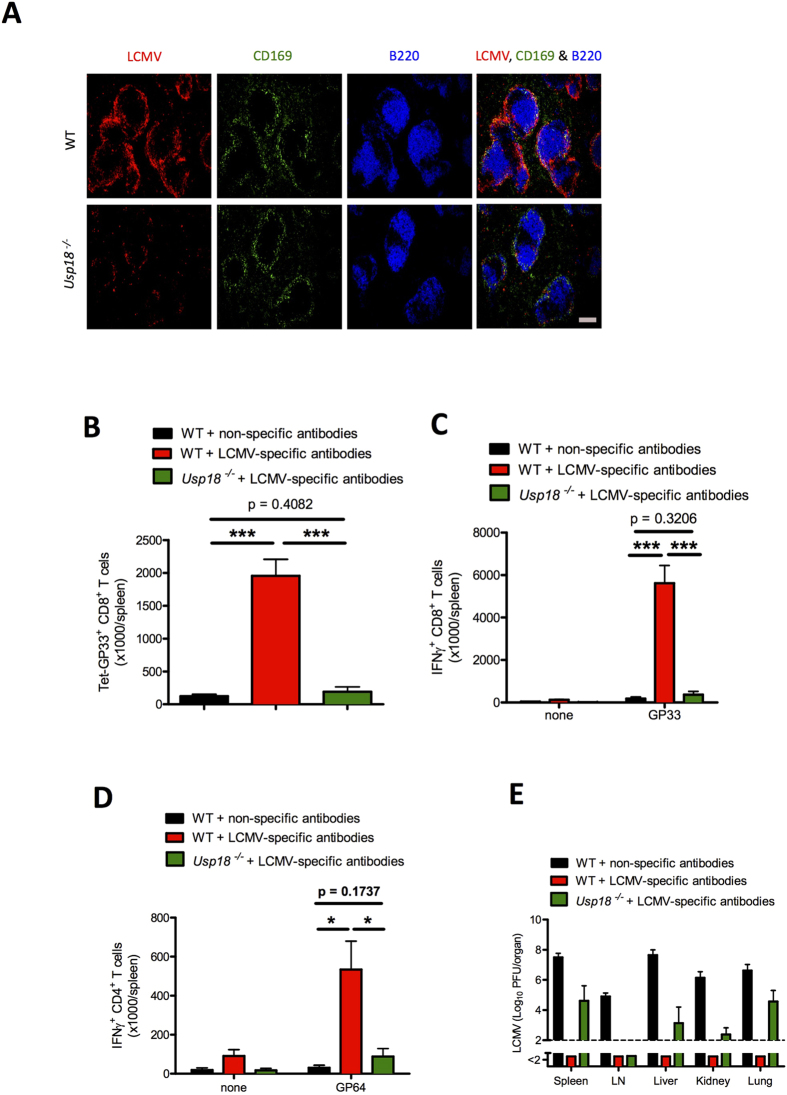
Immune activation in the presence of virus-specific antibodies is *Usp18* dependent. Lymphocytic choriomeningitis virus (LCMV)-specific antibodies were injected into *Usp18*^−/−^ mice and littermate control mice. Non-specific antibodies were injected into littermate control mice to form a control group. Mice were challenged with LCMV-Docile. (**A**) Immunohistochemical analysis of spleen showing LCMV nucleoprotein (red), marginal zone macrophages (CD169, green), and follicular B cells (B220, blue) after 1 day of infection with 2 × 10^6^ plaque-forming units (PFU) of LCMV-Docile (n = 3). Scale bar, 200 μm. (**B–E**) Mice were infected with 2 × 10^4^ PFU of LCMV-Docile and were evaluated for various parameters after 10 days of infection. (**B**) Total number of LCMV-specific T cells in the spleen that were positive for the MHC class I tetramer of the glycoprotein of LCMV (Tet-GP33^+^) and for CD8 (CD8^+^) (n = 5–8). (**C**) Total number of interferon (IFN)-γ^+^ CD8^+^ T cells in the spleen was determined after *in vitro* stimulation with or without LCMV GP33 peptide for 5 hours (n = 5–8). (**D**) Total number of IFN-γ producing CD4^+^ T cells after *in vitro* stimulation with or without LCMV GP64 peptide for 5 hours in spleen (n = 5–8). (**E**) Viral titers from spleen, inguinal lymph nodes (LN), liver, kidneys, and lungs (n = 7–8). Data are shown as mean ± SEM and are pooled from 2 or 3 independent experiments. *P < 0.05; **P < 0.01; ***P < 0.001 (Student’s *t*-test).

Furthermore, to examine the role of *Usp18* we transferred virus-specific antibodies to WT and *Usp18*^−/−^ mice and infected the mice with LCMV-Docile. In absence of *Usp18*, viral replication was impaired in splenic marginal zone macrophages (Fig. 8A and supplementary Figure 5). The absence of *Usp18* limited the expansion of virus-specific CD8^+^ T cells (Fig. 8B) and reduced IFN-γ production by CD8^+^ T cells (Fig. 8C) and CD4^+^ T cells (Fig. 8D). The lack of innate and adaptive immune activation in the absence of *Usp18* was associated with a problem in viral clearance (Fig. 8E). This finding suggests that, in the presence of virus-specific antibodies, *Usp18* is necessary for viral replication in marginal zone macrophages and also enhances the priming of virus-specific CD8^+^ T cells.

## Discussion

In the study reported here, we found that virus-specific antibodies limit the quantity of infectious virus in peripheral organs but still allow viral replication in the marginal zone. This specific distribution of virus after challenge with infection is beneficial for innate and adaptive immune activation; it limits immunopathology and leads to viral control.

Currently the World Health Organization (WHO) recommends 10 antiviral vaccinations, which clearly protect the vaccinated host from infection with the live pathogen. Natural viral infection induces plasma cells, which produce virus-specific antibodies^19^. Although specific antibodies mainly target infectious virus particles, virus-specific CD8^+^ T cells can directly suppress viral replication in infected cells^20^. Currently available vaccinations induce measurable antibodies against the pathogen but often fail to induce virus-specific CD8^+^ T cells^21^. Some reports suggest that, after secondary infection or vaccination, contact with the virus will further activate the immune system, which then induces protective T-cell immunity^22^,^23^,^24^. Indeed, in the Friend virus model, antibodies can enhance the virus-specific CD8^+^ T-cell response^25^. The results of our study show that specific antibodies block viral replication within peripheral organs within the first days after infection but still allow enforced viral replication in lymphoid organs. We found that the presence of antibodies limited viral replication in the liver and induced more-efficient antiviral CD8 T cells. The organ-specific antiviral capacity of virus-specific antibodies may be due to differential expression of Fc receptors in different organs. One way to induce such antiviral mechanisms could be via Fc receptor III (CD16), which either tracks virus into various vesicular compartments or induces antiviral activity^26^. In addition, complement may track virus into a separate compartment^27^. Further studies remain to be done to analyse the mechanism of Fc receptors on antiviral activity in macrophages. The production of antiviral cytokines and virus-specific CD8^+^ T cells could be induced and could lead to rapid control of recall infection. Therefore, we suggest that antibodies are, at least for some viruses, much more potent memory components than are CD8^+^ T cells.

Immunological memory against hepatitis C virus (HCV) is a challenge to the immune system, and the generation of vaccines against HCV has failed to date^28^. One reason for this failure is that the virus can mutate quickly during infection; when this happens, the existing immunological memory is no longer protective^29^. On the basis of our findings, we suggest that a good vaccine should still allow some viral replication in certain secondary lymphoid niches but should also inhibit the spread of virus to the susceptible organ. We suggest that, because HCV replication in antigen-presenting cells is very limited or almost absent, it is probably impossible for CD8^+^ T cells to be primed in the presence of virus-specific antibodies. This hypothesis may at least partially explain the failure of HCV vaccines. Recently it was shown that preexposure to HCV antigen induces the production of CD8^+^ T cells, which suppress the immune response after viremic infection with HCV^30^. Although the authors of that study explained this phenomenon by the presence of regulatory T cells, it is possible that rapid inhibition of viral replication may limit the draining of HCV antigen to secondary lymphoid organs and can thereby limit the induction of a protective antiviral immune response.

Of course, we may question whether the mechanisms we found for LCMV are also relevant to HIV. HIV-specific antibodies do not induce protective immunity^31^, and cellular immunity does not lead to a protective immune response upon challenge infection^32^. The fact that HIV induces marginal zone atrophy and marginal zone lymphoma^33^,^34^ and the fact that CD4^+^ T cells can be activated in the marginal zone and are the main target cells of HIV infection^14^ suggest that the marginal zone is a niche in which activated CD4^+^ T cells are easily infected. In light of our findings, we suggest that allowing HIV replication in the marginal zone of the spleen may in this special case be a disadvantage, because activated CD4^+^ T cells are located mainly within the marginal zone. Although this hypothesis could explain the difficulties in generating an HIV vaccine^31^, the generation of more data from HIV animal models is necessary before we can draw conclusions about the relationship between antibodies, CD8^+^ T cells, and HIV replication in the marginal zone.

In conclusion, we found that specific antibodies are much more potent than CD8^+^ T cells in protecting mice against viruses that are prone to persistence because these antibodies blunt the replication of virus in peripheral organs but allow replication of virus in the marginal zone, thereby leading to effective immune priming.

## Methods

### Mice

CD169-DTR, *Jh*^−/−^*, Prf1*^−/−^, and *B2m*^−/−^ mice were maintained on a C57BL/6 background. CD45.1 congenic mice were used as wild type mice to track the cells. *Usp18*^−/−^ mice were maintained on a mixed background, and mice were directly compared to littermate controls. KL25 mice, which express the immunoglobulin heavy chain of LCMV-neutralizing antibodies were maintained on a CD45.1 background and serum of LCMV infected KL25 mice was used as positive control for *in vitro* virus neutralization assay. This study was approved by the Nordrhein Westfalen Landesamt für Natur, Umwelt und Verbraucherschutz (Recklinghausen, Germany) and carried out in accordance with the German law for animal protection. All the experimental protocols were approved by the Nordrhein Westfalen Landesamt für Natur, Umwelt und Verbraucherschutz (Recklinghausen, Germany) or with the institutional guidelines of the Ontario Cancer Institute of the University Health Network and at McGill University.

### Pathogens and plaque assays

The LCMV-WE and LCMV-Docile strains were originally obtained from F. Lehmann-Grube (Heinrich Pette Institute, Hamburg, Germany) and were propagated on L929 cells, MC57 cells, or both. Mice were infected intravenously with various doses of LCMV. LCMV viral titers were detected by plaque-forming assays on MC57 fibroblasts, as previously described^35^. A replication-deficient recombinant LCMV (rLCMV; kindly provided by Pinschewer) that express the mutated form of LCMV-GP but still has antigenic properties was produced according to standard protocols^36^ and was injected intravenously into mice. The recombinant *Listeria monocytogenes* expressing the epitope of glycoprotein 33–41 of LCMV (LM-GP33) and wild-type *Listeria monocytogenes* (LM-WT) were grown in brain-heart infusion medium diluted in phosphate buffered saline (PBS) and were injected intravenously into mice.

### Memory cells and immune serum isolation and transfer

Six- to 8-week-old C57BL/6 naïve mice were infected intravenously with 2 × 10^6^ PFU of LCMV-WE. After 80 to 120 days of infection, immune components were isolated from these memory mice. LCMV-immune serum was collected and pooled from a group of mice, and virus-free serum was used to inject intravenously into mice for all experiments. CD8^+^ T cells, CD4^+^ T cells, and B220^+^ cells were isolated from spleen of memory mice with magnetic-activated cell sorting (MACS) isolation kit, according to the manufacturer’s protocol (Miltenyi Biotec, Germany). Serum and memory-cell transfers were performed 2 days before the infection. Mice were injected once intravenously with 300 μl of immune serum, 5 × 10^6^ memory CD8^+^ T cells, 1 × 10^7^ memory CD4^+^ T cells, or 1 × 10^7^ memory B cells. *Prf1*^−/−^ mice were infected with 2 × 10^5^ PFU of rLCMV and for control group C57BL/6 naïve mice got 2 × 10^5^ PFU of rLCMV infection. After 30 days spleens of these memory mice were used as donors for LCMV-specific memory CD8^+^ T cells.

### Histologic analysis

Histologic analyses of snap-frozen tissues were performed with mouse monoclonal antibodies to LCMV nucleoprotein (NP; made in house), CD169 (MCA884F; AbD Serotec, Germany), or CD45R/B220 (RA3-6B2; eBioscience, Germany). Red pulp macrophages were stained with F4/80 (BM8; eBiosciences).

### Enzyme-linked immunosorbent assays

Interferon-alpha (IFN-α) enzyme-linked immunosorbent assays (ELISA) were performed according to the manufacturer’s protocol (PBL Interferon Science, Germany).

### Flow cytometry

The Tetramer Facility of National Institutes of Health (NIH) provided LCMV-GP33 tetramer. Cells were stained with allophycocyanin (APC)-labeled GP33 MHC class I tetramer (GP33/H-2Db) for 15 minutes at 37 °C. After incubation, the samples were stained with anti-CD8 (clone 53–6.7; eBioscience) or anti-CD4 (clone GK1.5; eBioscience) antibodies for 30 minutes at 4 °C. Absolute numbers of GP33-specific CD8^+^ T cells were calculated with fluorescent beads (BD Biosciences) by using fluorescence-activated cell sorting (FACS). For measurement of intracellular IFN-γ, cells were fixed with 2% formaldehyde in PBS for 10 minutes, permeabilized with 1% saponin in FACS buffer at room temperature, and stained with anti–IFN-γ antibody for 30 minutes at 4 °C (clone XMG1.2; eBioscience). All stained cells were analysed with a FACSFortessa (BD Biosciences) flow cytometer, and data were analysed with FlowJo software.

### ALT and LDH measurement

The activity of ALT and LDH was measured in the Central Laboratory, University Hospital Essen, Germany.

### LCMV neutralization assay

The neutralizing capability of serum was measured with plaque-forming assays according to a previously published protocol^35^.

### Statistical analysis

Data are expressed as means ± SEM. Student’s *t*-test was used to detect statistically significant differences between groups. Significant differences between several groups were detected by one-way analysis of variance (ANOVA) with the Bonferroni or Dunnett post hoc test. The level of statistical significance was set at *P* < 0.05.

## Additional Information

**How to cite this article**: Duhan, V. *et al.* Virus-specific antibodies allow viral replication in the marginal zone, thereby promoting CD8^+^ T-cell priming and viral control. *Sci. Rep.* **6**, 19191; doi: 10.1038/srep19191 (2016).

## Supplementary Material

Supplementary Information
