# Supplementary material for: Virus-specific antibodies allow viral replication in the marginal zone, thereby promoting CD8+ T-cell priming and viral control
Source: Sci Rep. 2016 Jan 25;6:19191. doi: 10.1038/srep19191 (PMC4726415; doi:10.1038/srep19191)
Supplement: Supplementary Information [file srep19191-s1.pdf]

Virus-specific antibodies allow viral replication in the marginal zone, thereby promoting CD8+ T-cell priming and viral control

Vikas Duhan[1,7], Vishal Khairnar[1,7], Sarah-Kim Friedrich[1], Fan Zhou[1], Asmae Gassa[1], Nadine Honke[1], Namir Shaabani[1], Nicole Gailus[1], Lacramioara Botezatu[2], Cyrus Khandanpour[2], Ulf Dittmer[3], Dieter Häussinger[4], Mike Recher[5], Cornelia Hardt[1], Philipp A. Lang[4,6,7] and Karl S. Lang[1,4,7,*]

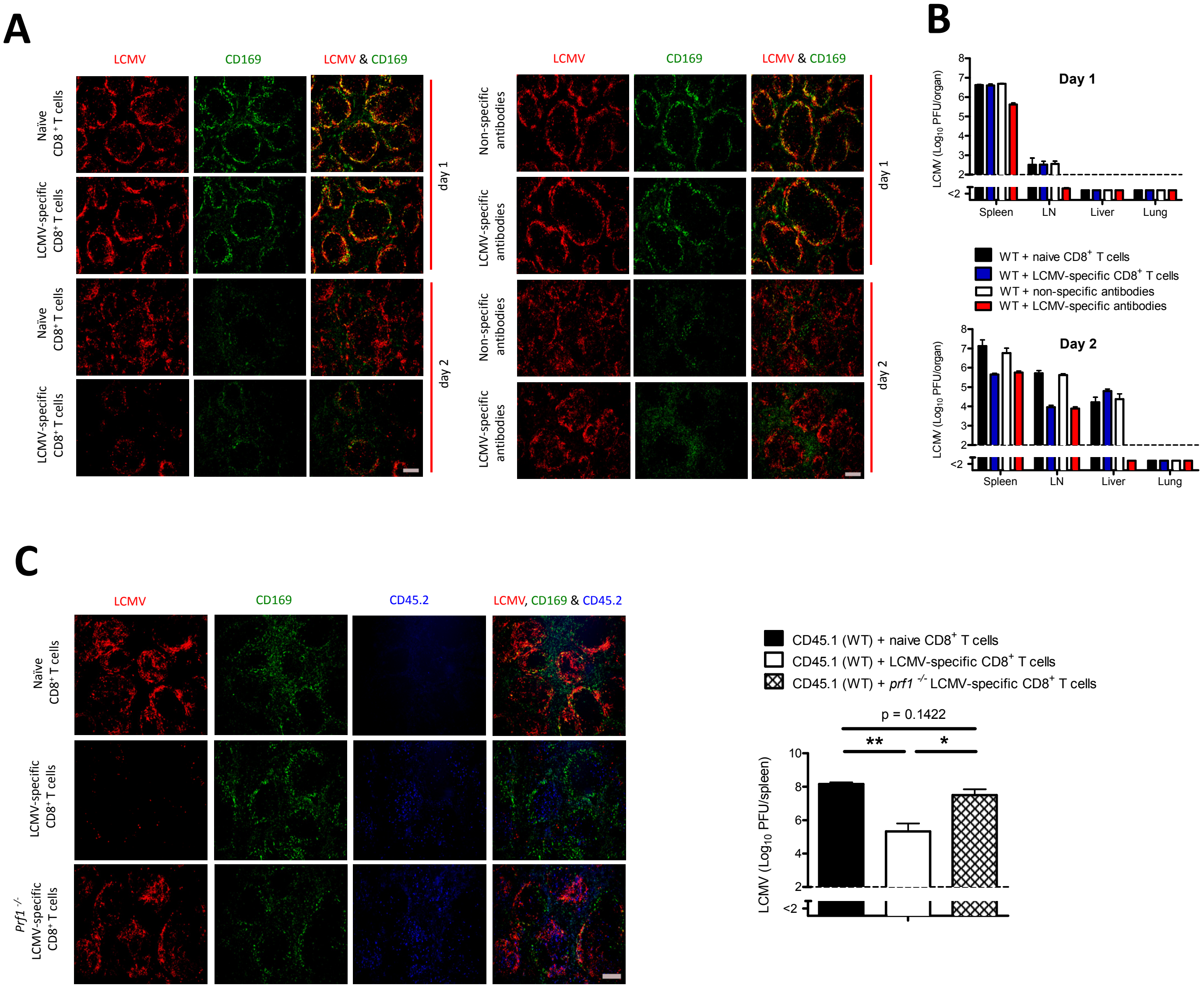

Supplementary Figure 1: Virus-specific antibodies, but not virus-specific CD8+ T cells, allow viral replication in the marginal zone. (A, B) C57BL/6 naïve mice were injected with naïve CD8+ T cells, non-specific antibodies (naïve serum), lymphocytic choriomeningitis (LCMV)-specific CD8+ T cells, or LCMV-specific antibodies (immune serum). After 2 days all mice were infected with 2×10^6 plaque-forming units (PFU) of LCMV strain WE (LCMV-WE). (A) Representative immunofluorescence of spleen is shown after day 1 and 2 of infection, stained for LCMV nucleoprotein (red) and marginal zone macrophages (CD169, green). One slide representative of 3 slides is shown. (B) Viral titers from spleen, inguinal lymph nodes (LN), liver, and lungs after 1 or 2 days of viral infection (n = 3). (C,D) Memory CD8+ T cells isolated from C57BL/6 memory and *prf1* $^{-/-}$ memory mice were injected to CD45.1 (wild-type; WT) naïve mice. One CD45.1 (wild-type; WT) group received naïve CD8+ T cells as control. After 2 days mice were injected with 2×10^4 PFU of LCMV-WE.(C) Representative immunofluorescence of the spleen is shown after 3 days of infection, stained for LCMV nucleoprotein (red), marginal zone macrophages (CD169, green) and transferred CD8+ T cells (CD45.1, blue). One slide representative of 3 experiments is shown. (D) Graph shows the viral titers in the spleen after 3 days of infection. Horizontal dotted lines designate the detection limit. Data are shown as mean ± SEM. *$P < 0.05$; **$P < 0.01$; ***$P<0.001$ (Student's *t*-test).

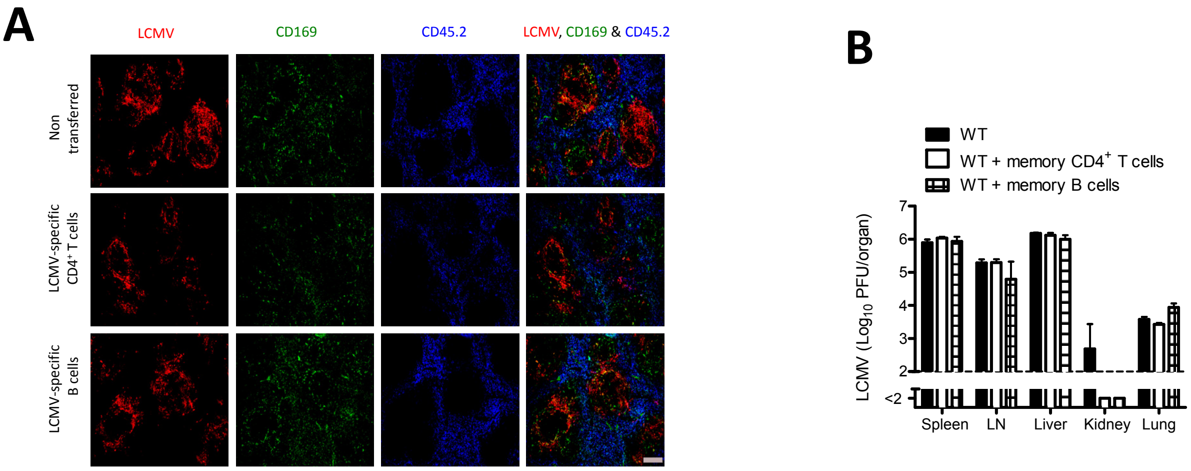

Supplementary Figure 2: Memory CD4+ T cells and memory B cells has no effect on viral replication in the marginal zone.
C57BL/6 naïve mice were injected with memory B220+ B cells and memory CD4+ T cells isolated from memory mice. One group of mice was left untreated. After 2 days all mice were infected with 2×10^6 plaque-forming units (PFU) of lymphocytic choriomeningitis virus strain WE (LCMV-WE). (A) Representative immunofluorescence in spleen after 3 days of infection, stained for LCMV nucleoprotein (red), marginal zone macrophages (CD169, green), and red pulp macrophages (F4/80, blue). One representative slide of 3 is shown. (B) Viral titers from various organs after 3 days of viral infection (n = 3). Horizontal dotted lines designate the detection limit. Data are shown as mean ± SEM.

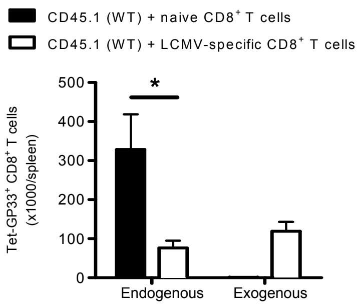

Supplementary Figure 3: Memory CD8$^+$ T cells reduce the expansion of endogenous CD8$^+$ T cells.
CD45.1 (wild-type; WT) congenic naïve mice were injected with lymphocytic choriomeningitis virus (LCMV)-specific CD8$^+$ T cells isolated from C57BL/6 memory mice. One group of mice was injected with naïve CD8$^+$ T cells isolated from naïve C57BL/6 mice. After 2 days all mice were infected with 2×10^6 plaque-forming units (PFU) of LCMV strain WE (LCMV-WE). Total numbers of endogenous and exogenous T cells positive for the MHC class I tetramer of the glycoprotein of LCMV (Tet-GP33$^+$) and for CD8 (CD8$^+$) in the spleen were determined after 10 days of infection (n = 3-4). Data are shown as mean ± SEM. *$P < 0.05$ (Student's *t*-test).

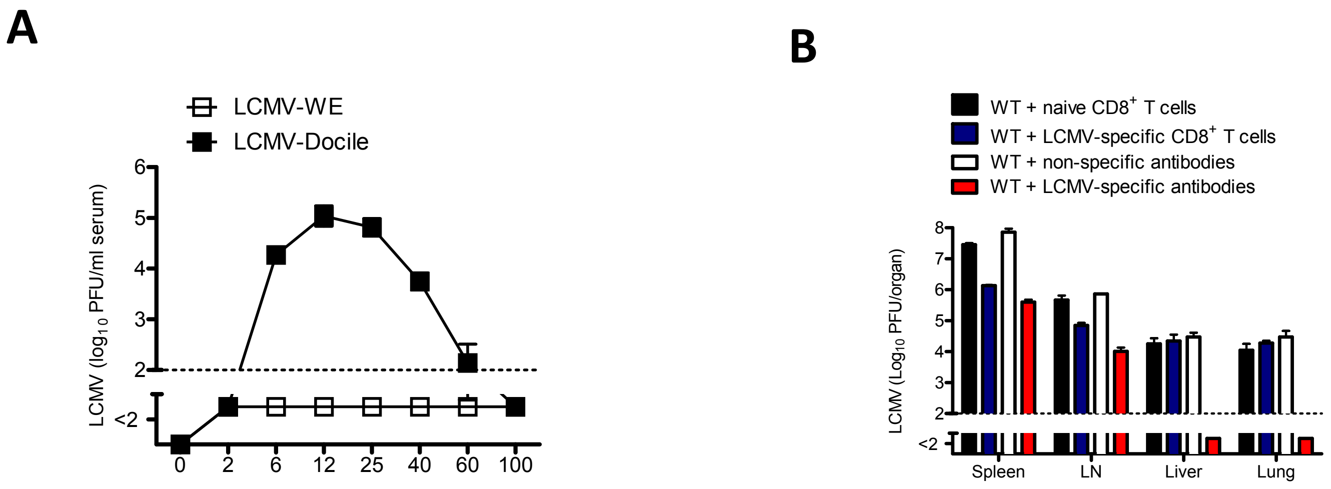

Supplementary Figure 4: Virus-specific antibodies inhibit persistent LCMV-Docile replication in peripheral organs.

(A) C57BL/6 naïve mice were infected separately with 2×10^4 plaque-forming units (PFU) of lymphocytic choriomeningitis virus strain WE (LCMV-WE) and LCMV-Docile. Graph shows viral titers in serum on indicated days. (B) C57BL/6 naïve mice were injected with naïve $CD8^+$ T cells, non-specific antibodies (naïve serum), LCMV-specific $CD8^+$ T cells, or LCMV-specific antibodies (immune serum). After 2 days all mice were infected with 2×10^4 PFU of LCMV-Docile. Graph shows viral titers in spleen, inguinal lymph nodes, liver, and lungs after 3 days of infection. Horizontal dotted lines designate the detection limit. Data are shown as mean ± SEM

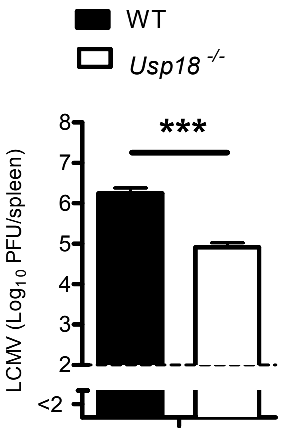

Supplementary Figure 5: *Usp18* promotes LCMV replication.

Usp18$^{-/-}$ mice and littermate control naïve mice (wild-type; WT) were infected with 2×10^{6} plaque-forming units (PFU) of lymphocytic choriomeningitis strain Docile (LCMV-Docile). Graph shows viral titers in spleen after 1 day of infection (n = 4). Horizontal dotted lines designate the detection limit. Data are shown as mean ± SEM. ***P < 0.001 (Student's *t*-test).
